# Postoperative Multimodal Analysis in Successful Gas Displacement of a Submacular Hemorrhage

**DOI:** 10.1155/2021/5577826

**Published:** 2021-06-03

**Authors:** Tatiana Urrea-Victoria, Emiliano Fulda-Graue, Miguel A. Quiroz-Reyes, Felipe Esparza-Correa, Alejandra Nieto-Jordan, Erick A. Quiroz-Gonzalez, Federico Graue-Wiechers

**Affiliations:** Instituto de Oftalmología Fundacíon Conde de Valenciana (Non-profit Organization), Chimalpopoca 14, Colonia Obrera, Mexico City 06800, Mexico

## Abstract

In this report, we describe a case of timely gas vitrectomy to displace a moderate submacular hemorrhage from the submacular space without tPA, release vitreoretinal traction along the borders of a posterior retinal tear, and analyze postoperative multimodal imaging findings in a 34-year-old male patient whose right eye was injured by a stone. The patient underwent a successful nontissue plasminogen activator gas vitrectomy 3 days after the accident. A multimodal evaluation with spectral-domain optical coherence tomography (SD-OCT), 10-2 and 30-2 campimetry, microperimetry, multifocal electroretinography (mfERG), and visual evoked potentials was performed 6 months after the accident. The multimodal imaging tests yielded abnormal foveal SD-OCT patterns, with a fibrous sealed tear in the retinal pigment epithelium. Campimetry showed low levels of retinal sensitivity; microperimetry and mfERG revealed a subnormal retinal response and a reduction in the N1 and P1 wave amplitudes. The visual evoked potential responses were normal. Multidisciplinary examination at 6 months postoperatively revealed a structurally and functionally abnormal macula. The retina remained attached. Our functional findings indicate that submacular hemorrhage should be treated in a timely manner to minimize photoreceptor damage.

## 1. Introduction

Closed-globe blunt ocular trauma mechanisms can rupture the vascular choroid and result in central vision-threatening submacular hemorrhage (SMH) [1]. This condition is defined as the presence of a significant hemorrhage under the neurosensory macula [2], which is generally caused by the contusional breakdown of the blood–retina barrier [3]. Besides ocular trauma, the most common conditions that may cause an SMH are pathological myopia, pathological submacular choroidal neovascularization, polypoidal choroidal vasculopathy, arterial macroaneurysms, and presumed ocular histoplasmosis syndrome [2, 4]. Blunt ocular trauma with SMH is a major cause of severe vision loss [4], usually affecting young patients and causing several pathological conditions such as commotion retinae, choroidal rupture, macular hole, subretinal and macular hemorrhage, retinal vascular rupture, vitreous hemorrhage, retinal pigment epithelial edema and healed scarring, retinal dialysis, macular infarction, traumatic optic neuropathy, optic nerve avulsion [2], and even retinal tears. Clinical and histopathological studies have shown that prolonged contact of the blood catabolic products with photoreceptors in the subretinal space worsens the prognostic and visual outcomes [5].

Postcontusional choroidal rupture usually runs vertical and temporal to the optic nerve disc and sometimes runs through the foveal center, with different and significant amounts of secondary hemorrhagic macular detachment in 66% of cases, resulting in poor visual outcomes, particularly when the SMH persists for several days [2, 3].

Here, we describe the case of a patient with a trauma-related SMH complicated with a large tractional posterior paramacular retinal tear; these two posttraumatic lesions were not connected or tunneled together at the level of the subretinal space since this would have facilitated its simultaneous surgical approach to treat this condition only with gas vitrectomy pneumatic displacement of the blood and release of the vitreoretinal traction complemented with laser retinopexy, without the use of tissue-plasminogen activator (tPA) to avoid additional potential retinal toxic damage. We would like to emphasize the efficacy of our simplified surgical approach and the importance of timing in surgical intervention to achieve adequate anatomical and functional recovery with minimal sequalae at the subclinical level as demonstrated by the multimodal imaging structural and functional evaluations.

## 2. Case Presentation

The Retina Department at the *Instituto de Oftalmologia* Hospital, Mexico City, Mexico, provided authorization and released the electronic clinical record pertaining to this case report. This study adhered to the tenets of the Declaration of Helsinki and received approval from the institutional ethics committee (no reference number is usually provided for a case report by this institution). Written informed consent was obtained from the patient in accordance with the institutional guidelines. Data are available from the imageology and psychophysics laboratory at the Retina Department of the Institution.

**Figure 1:**
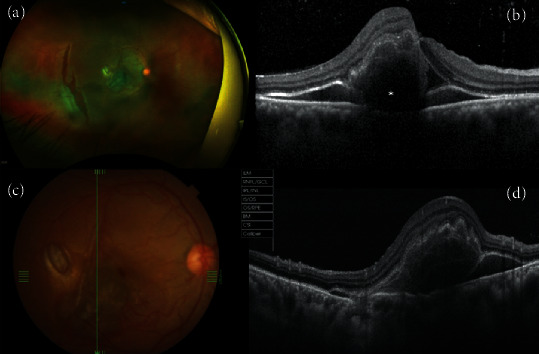
(a) A wide-field preoperative photograph shows limited moderate submacular hemorrhage associated with a parafoveal choroidal rupture and an irregular-shaped posterior retinal tear. (b) A swept-source cross-sectional optical coherence tomography (OCT) image in the horizontal plane shows a submacular hemorrhage that appears hyperreflective; it is hemoglobin-rich (^∗^) and hyporeflective when the serous component is predominant. Due to the presence of blood, there is attenuation of the IS/OS (inner segment/outer segment) band and RPE (retinal pigment epithelium) line. (c) Posterior pole preoperative clinical photography and (d) corresponding swept-source cross-sectional OCT image in the vertical plane depict an elevated preoperative central submacular hemorrhage and an RPE attenuation-like defect.

A 34-year-old man presented with reduced central vision in his right eye 3 days after experiencing blunt trauma due to being struck by a stone. The preoperative best-corrected visual acuity (BCVA) was 1/200 in the affected eye. The intraocular pressure was 10 mmHg, and there was a pupillary sphincter rupture. On biomicroscopy assessment, the vitreous cavity showed partial posterior detachment with vitreoretinal traction around the edges of an irregular posterior retinal tear, with a very shallow subretinal fluid-like appearance and neuroretinal whitening around the tear. The rest of the retina was completely attached, and no communication between the posterior retinal tear and the SMH was documented on optical coherence tomography (OCT). The macula was dome-shaped and elevated because of the presence of a moderately thick SMH (Figures 1(a)– 1(d)). Cross-sectional images (Figures 1(b) and 1(c)) were acquired along the horizontal and vertical planes through the foveal center using a Swept-Source DRI Optical Coherent Tomography Triton device (Topcon Medical Systems, Inc., Oakland, NJ). An SS-OCT image crossing the retinal tear was not obtained.

Although only injection of an expansible gas bubble was considered the finding of a large posterior tear in the relatively young man, with traction of a nonliquefied vitreous cortex around the edges of the tear, allowed us to decide on the use of surgery as treatment modality. It is important to emphasize that it was not possible to drain the submacular hemorrhage through the retinal tear by avoiding concomitant retinal detachment. This would have facilitated the total transoperative removal of the submacular blood on the third day, when surgery was performed. However, it was decided not to use tPA because of its potential retinal toxicity, leaving only gas followed by strict postoperative prone position to achieve the pneumatic displacement of the blood.

Under local anesthesia and sedation, the patient underwent a pneumatic submacular blood displacement procedure, which involved a gas pars plana vitrectomy and the release of vitreoretinal traction around the retinal tear. Subsequently, central vitrectomy was performed with removal of the cortical vitreous body from the retinal surface, using a silicone-tipped cannula and active suction. The surgeon was careful to obtain a free and mobile posterior hyaloid membrane. Laser retinopexy was performed around the retinal tear, and an air-fluid exchange was conducted at the end of the surgical procedure using a mixture of nonexpandable 10% perfluoropropane gas (C_3_F_8_). Postoperatively, the patient was instructed to strictly remain in the prone position for 5 days and follow the protocol for recovery recommended for this type of blunt trauma treated with pneumatic submacular blood displacement. Local and systemic antibiotics were administered after the surgical procedure.

**Figure 2:**
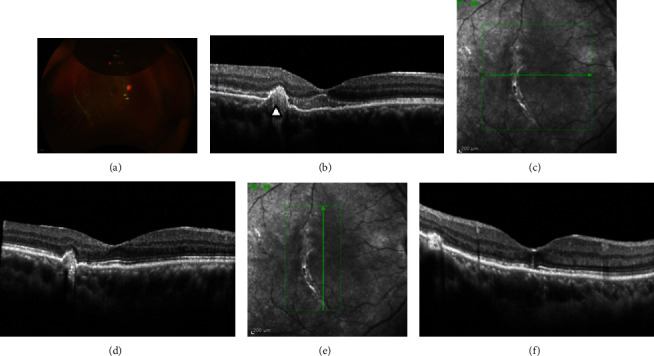
(a) Six weeks after surgery. The 20% remaining perfluoropropane (C_3_F_8_) bubble with a posterior temporal retinal tear treated with laser. (b) The 6-week spectral-domain optical coherence tomography (SD-OCT) corresponding to the submacular hemorrhage is almost completely reabsorbed. The IS/OS and external limiting membrane (ELM) lines show irregularity and are compacted against the protruded hyperreflective retinal pigment epithelium (arrowhead). The retinal pigment epithelium shows bulging without rupture but only mild disruption and fused IS/OS with the ELM (c, d). The 6-month postoperative cross-sectional horizontal and (e, f) vertical planes through the foveal center on SD-OCT.

**Figure 3:**
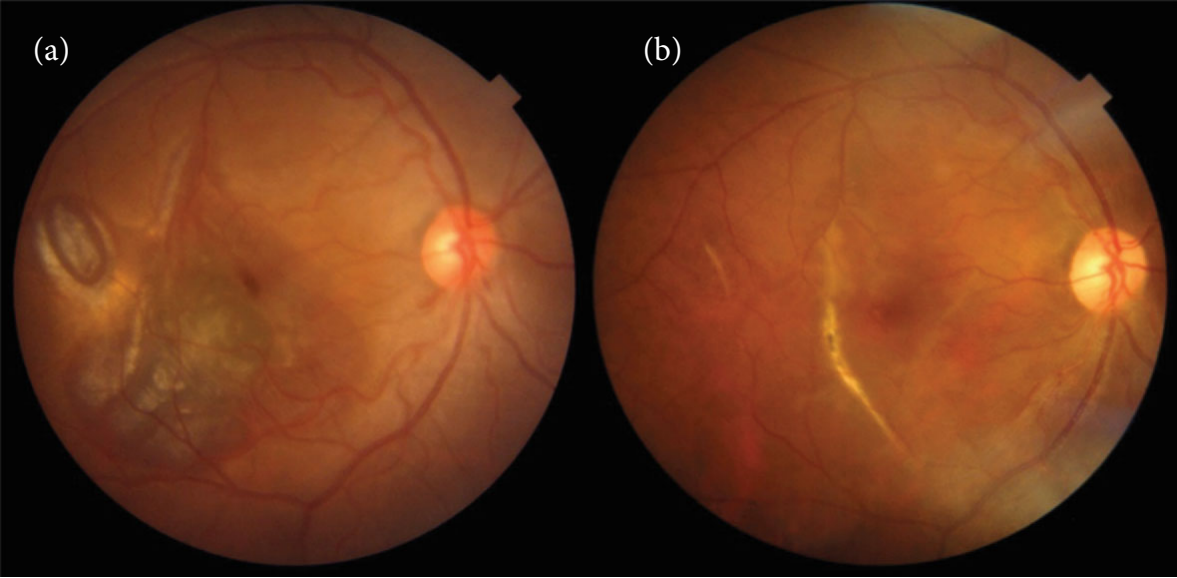
Preoperative and 6-month postoperative fundus color photographs. (a) Submacular hemorrhage associated with an adjacent parafoveal choroidal rupture. (b) Six months after surgery: a clinically normal foveal appearance and subretinal reparative scarring along the choroidal rupture temporal to the fovea.

**Figure 4:**
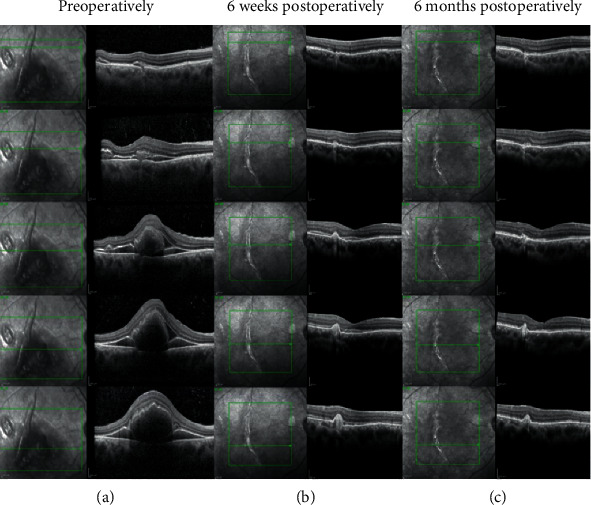
Preoperative spectral-domain optical coherence tomography (SD-OCT) of the corresponding macular locus images. Preoperative SD-OCT (left vertical column), 6-week postoperative SD-OCT (central vertical column), and 6-month postoperative comparative SD-OCT (right vertical column). (a–c) SD-OCT cross-sectional images acquired along the horizontal plane through the foveal center depict the involuted presence of blood and the sequential slow improvement of the retinal pigment epithelium, IS/OS, and external limiting membrane layers.

At the 6-week follow-up visit (Figures 2(a) and 2(b)), the blood had been inferiorly displaced, the BCVA was 20/140, eye pressure was normal, the retina was attached with some blood remnants inferiorly, and the macula had flattened, without underlying blood remnants.  Figure 3 shows that the BCVA was 20/30, the medium was very clear, the retina was attached, and the posterior retinal tear had a tight seal with the retinal pigment epithelium (RPE). Postoperative multimodal imaging analysis was performed to obtain the structural and functional patterns such as BCVA, visual field testing evaluation, and multifocal electroretinography, and microperimetry evaluation. The postoperative spectral-domain optical coherence tomography (SD-OCT-Spectralis OCT, Heidelberg Engineering, Germany) pattern showed a resolved submacular hemorrhage, an ellipsoid (IS/OS) line, and external limiting membrane (ELM) SD-OCT biomarker lines preserved but distorted and fused with the protruding RPE line (Figure 4).

**Figure 5:**
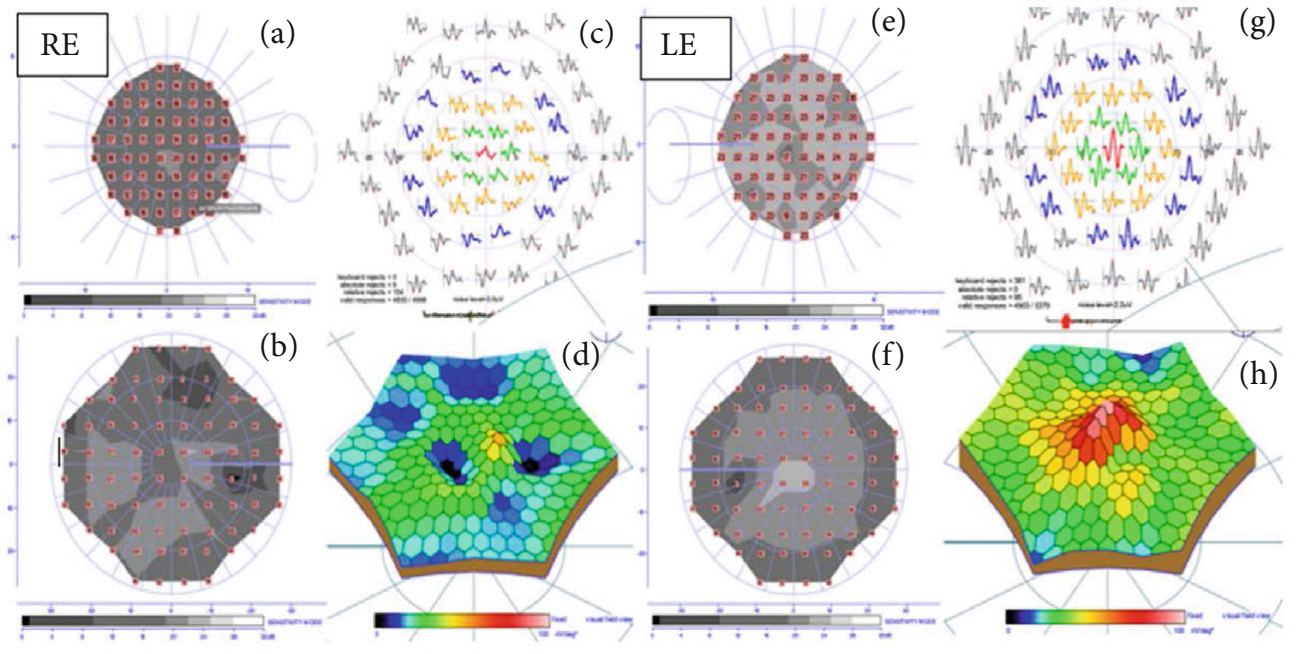
Comparative campimetric and multifocal electroretinography (mfERG). Right eye (a) 10-2 and (b) 30-2 visual field view testing show deficits in retinal sensitivity to the central points of the stimuli. The mean deficits are 9.0 and 7.8 dB, respectively, compared with the contralateral normal eye (normal mean deficit: 0.5-2.0 dB). (c) Subnormal retinal response and reduction of the N1 and P1 wave amplitudes. The 61-hexagon 30° test had decreased by 75.0%, 41.9%, 27.0%, 10.5%, and 6.15%, respectively, from the <2-degree to >15-degree central rings compared to the normal control parameters (normal range of the mean deficit permitted is 1-3%). (d) mfERG as a 3-D topographic field map of the macular function shows subnormal foveal photoreceptor sensitivity with a normal spatial resolution, due to stable fixation and location pattern. Left eyes comparative normal figures of the left eye show (e) 10-2, (f) 30-2, and (g, h) mfERG within the normal range.

Figures 5(a) and 5(b) show the 10-2 and 30-2 automated central field testing (Visual Field Perimeter, Vision Monitor MonPackONE, MetroVision, France), which revealed mean deficits in the retinal sensitivity of 9.0 dB and 7.8 dB, respectively, at the central points of the stimuli. No other visual field changes were detected.

Figures 5(c) and 5(d) show that the N1 wave amplitude of the multifocal electroretinography (mfERG) (Electrophysiology Vision Monitor Analyzer MonPackONE, MetroVision, France). A 61-hexagon 30° test had decreased by 75.0%, 41.9%, 27.0%, 10.5%, and 6.15%, respectively, from the <2-degree to >15-degree central rings (normal range of the mean deficit allowed is 1-2%). The implicit time was shorter in the <2-degree central ring and slightly longer in the remaining central rings. The nV amplitude of the P1 waves significantly decreased with subnormal implicit times in all central rings studied in the affected eye, compared to the normal contralateral eye.  Figure 5(d) shows the mfERG as a 3-D topographic field map of the macular function that shows subnormal foveal photoreceptor sensitivity with a normal spatial resolution due to stable fixation and location pattern.

**Figure 6:**
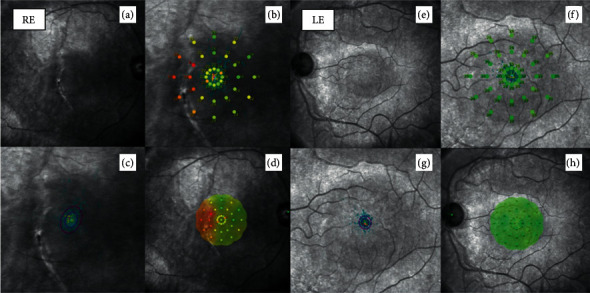
Comparative 6-month microperimetry. (a) The clinical aspect depicts a normal central fovea with a juxtafoveal choroidal rupture. (b) Retinal sensitivity measured with a microperimetry device (MAIA) shows a very diminished retinal sensitivity temporal to the fovea and an abnormal response 6 months following the surgery. The red figures and numbers indicate abnormally low retinal sensitivities (dB). (c) Stable foveal fixation pattern of the affected eye. (d) Corresponding foveal plotting with low retinal sensitivity on the temporal side of the fovea. (e–h) Correspond to normal left eye microperimetry.

**Figure 7:**
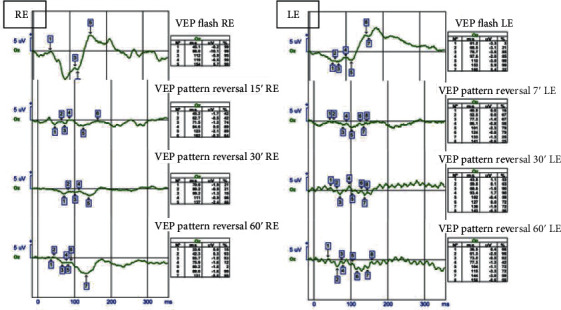
Visual evoked potentials. Comparative visual evoked potentials of the affected eye. N75-74.115 ms, P100-100.225 ms, and P135 were not recorded. Normal left eye VEP functional traces were considered between the normal ranges.

On microperimetry (MP-3 MAIA Confocal Microperimetry, MetroVision, France), the fixation pattern was found to be stable, and the fixation location pattern was documented as foveocentral. The retinal sensitivity analysis map showed abnormal macular integrity with reduced sensitivity, mainly in the center of the fovea, as well as reduced sensitivity over the choroidal rupture. There was no correlation between the reduced retinal sensitivity locus and the corresponding retinal thickness on OCT (Figure 6). The visual evoked potentials (RETeval, LKC Technologies; Gaithersburg, MD) were measured with a midoccipital active scalp electrode placed (Oz) in the midline 3-4 cm above the inion, a reference electrode on the forehead, and the ground electrode placed on the earlobe where normal (Figure 7).

## 3. Discussion

Here, we have reported a case in which early treatment with gas vitrectomy yielded successful displacement of the submacular blood as well as release of vitreoretinal traction at the margins of the posterior tear. Moreover, a strict prone positioning regimen allowed the sealing of the tear, preservation of the retinal anatomical structure, and displacement of the submacular blood outside the central area of the macula; all the previous facts have also been demonstrated in other studies [1, 3, 4, 6]. The use of postoperative multimodal imaging analysis with serial SS-OCT, SD-OCT, mfERG, and microperimetry in this case highlights a possible clinical and functional contribution to the study and knowledge of this vision-threatening traumatic condition. Our analysis illustrated a point-to-point correlation between the disruption of the external layers of the retina (RPE, IS/OS band, and ELM line) and the reduced retinal sensitivity in the microperimetry and mfERG, as well as a good correlation between the SD-OCT microabnormalities in retinal thickness and the same retinal locus of reduced retinal sensitivity on microperimetry.

The main aspect to consider when managing this condition is rapid blood displacement [2]. The patient in our case only underwent gas vitrectomy with nonexpanding 10% C_3_F_8_ and received no tPA, which has been used previously [1, 6].

This patient showed choroidal rupture temporal to the fovea, similar to what was described in others studies [2], and did not show evidence of complications, such as choroidal neovascularization or subfoveal scarring (Figures 2(c)–2(f) and Figure 4(c)).

The natural course of SMH varies according to the etiology, duration, diameter, and thickness of the hemorrhage [7]. It has been reported that an SMH is a devastating complication of various pathological entities, and in the case of a large or massive SMH secondary to blunt trauma, the prognosis for visual recovery is usually poor, with the final BCVA ranging from 20/200 to light perception [8].

Numerous approaches have been described for the displacement of SMH, including vitrectomy with the use of intravitreal or subretinal recombinant tPA (r-tPA), intravitreal gas tamponade, postoperative positioning [4], and pneumatic displacement with or without tPAs [6]. Ultimately, the preferred technique is defined by the duration and the extent of the SMH in addition to the surgeon's skill and preference [9].

In 1991, Peyman et al. [10] first reported the visual outcomes of r-tPA-assisted SMH removal. In their study, 60% of patients demonstrated an improvement in BCVA by two or more lines. A less invasive procedure is described by Chen et al. [11] which comprised of an intravitreal injection of r-tPA and gas tamponade to facilitate lysis of the SMH. This procedure allows clinicians to avoid surgical intervention for draining an SMH [6]. Early studies used subretinal tPA and perfluorocarbon liquid to facilitate aspiration through a small retinotomy [6]. However, this technique has become less common because it is associated with poor postoperative visual acuity and a high rate of retinal detachment [6]. Balughatta et al. [5] recently reported a case series of C_3_F_8_ pneumatic displacement of limited traumatic SMH with excellent anatomical results and final visual acuities attributed only to gas injection and patient positioning, without surgery or the use of tPA.

In cases complicated by posterior retinal tears, we consider that the release of vitreoretinal traction is of paramount importance and the pneumatic displacement without tPA injection followed by a strict prone position, in cases complicated by posterior retinal tears such as the one reported here, is less invasive and potentially less toxic than the tPA-assisted surgical drainage of the SMH. The mechanism involves moving the blood away from the subfoveal space by using the long-acting gas buoyancy effect to displace the blood from the submacular foveocentral photoreceptors [1, 2, 4, 7, 11].

In this report, photoreceptor dysfunction was considered to be the result of blood pooling under the macula and contact with the central photoreceptors, although pneumatic displacement occurred within the first 3 days. We considered that the OCT changes in this case (Figures 1, 2, and 4(a)– 4(c)) were consistent with disruption of the photoreceptors and demonstrated by the loss of regularity and compacting of the IS/OS and ELM SD-OCT biomarkers with some degree of irregular fibrous RPE changes. Furthermore, the quantification of the differential threshold of retinal sensitivity or visual field testing performed under different stimuli conditions (both background and stimuli) allowed us to detect subtle abnormalities with 10-2 and 30-2 visual field examinations (Figures 5(a) and 5(b)). The mfERG showed outer retinal and photoreceptor abnormalities, but to optimize its value, it is important to compare the mfERG results with those of concurrent automated visual fields. The visual evoked potentials elicited were in the normal range, excluding traumatic optic neuropathy. The microperimetry findings showed a satisfactory correlation between the microstructural SD-OCT macular pathology and the corresponding functional abnormality determined by microperimetry, visual field testing, and mfERG. However, there was no correlation between the localized locus of reduced macular sensitivity and the corresponding retinal thickness on OCT (Figure 6). In this case, there were consistent changes in the microperimetry findings and deep abnormalities in the N1 wave assessed by the mfERG, which were indicative of localized outer retina and photoreceptor dysfunction, as previously noted.

We acknowledge the several inherent limitations and shortcomings of this case report. However, based on the analysis of our results as well as other findings reported on the literature, we can confirm that early non-tPA pneumatic displacement could be achieved without severe visual damage. Prospective and multicenter protocols are needed to improve the approach in severe or complicated cases of SMH, similar to the one reported here.

In summary, this report confirms that early removal of blood from beneath the macula within the first few days is important in cases of SMH. This case was complicated by the presence of a large posterior retinal tear and vitreoretinal traction, but vitreoretinal release and gas vitrectomy were sufficient to displace the submacular blood and maintain the retinal attachment with good anatomical and functional results. A few subclinical and consequently not significant abnormalities since they do not affect the quality of binocular vision were detected at the 6-month multimodal functional evaluation only with sophisticated diagnostic tools.

Our findings suggest that SMH should be treated as soon as possible to minimize photoreceptor damage. With early displacement of the hemorrhage, only subclinical damage ensues. We emphasize that if the blood stays in contact with the photoreceptors for a long time, the damage will be greater, with loss of vision and clinical sequelae. This report suggests retina surgeons to perform timely surgical techniques to allow displacement of blood from the submacular space and, thereby, open an innovative path to developing new drugs that could reduce the toxic effects of blood catabolism seen with the drugs currently in use.

## Data Availability

The photos, composite figures, and laboratory studies to support the findings of this study may be released upon written application to the Photographic, Psychophysics laboratory and Clinical Archives department at Instituto de Oftalmología Fundacíon Conde de Valenciana (Non-profit Organization), Chimalpopoca 14, Colonia Obrera, Mexico City 06800, Mexico and from the corresponding author upon request.
